# Landscape of Endometrial Cancer: Molecular Mechanisms, Biomarkers, and Target Therapy

**DOI:** 10.3390/cancers16112027

**Published:** 2024-05-27

**Authors:** Ioana-Stefania Bostan, Mirela Mihaila, Viviana Roman, Nicoleta Radu, Monica Teodora Neagu, Marinela Bostan, Claudia Mehedintu

**Affiliations:** 1 Filantropia Clinical Hospital, 011132 Bucharest, Romania; ioana-stefania.bostan@rez.umfcd.ro (I.-S.B.); claudia.mehedintu@umfcd.ro (C.M.); 2 Stefan S. Nicolau Institute of Virology, Center of Immunology, Romanian Academy, 030304 Bucharest, Romania; mirela.mihaila@virology.ro (M.M.); viviana.roman@virology.ro (V.R.); 3 Faculty of Pharmacy, Titu Maiorescu University, 040314 Bucharest, Romania; 4 Department of Biotechnology, University of Agronomic Sciences and Veterinary Medicine of Bucharest, 011464 Bucharest, Romania; nicoleta.radu@biotehnologii.usamv.ro; 5 Biotechnology Department, National Institute for Chemistry and Petrochemistry R&D of Bucharest, 060021 Bucharest, Romania; 6 Department of Immunology, ‘Victor Babes’ National Institute of Pathology, 050096 Bucharest, Romania; neagu.monica@gmail.com; 7 Faculty of Medicine, University of Medicine and Pharmacy Carol Davila, 050471 Bucharest, Romania

**Keywords:** endometrial cancer, molecular mechanisms, biomarkers, targeted therapy, immunotherapy

## Abstract

**Simple Summary:**

Endometrial cancer is a complex disease with multiple risk factors, distinct histological subtypes, and a variety of available therapeutic options. The disease’s pathogenesis involves several molecular mechanisms, such as genetic mutations, defects in the DNA mismatch repair (MMR) pathway, hormonal signaling pathway imbalances, epigenetic changes, and disturbances in angiogenesis. On the other hand, molecular biomarkers seem to have an essential role in the diagnosis, prognosis, prediction of therapeutic response, and monitoring of disease progression in endometrial cancer. This review highlights the most significant dysregulated molecular mechanisms and reveals the relationship between specific biomarkers and targeted therapy approaches in the fight against endometrial cancer.

**Abstract:**

Endometrial cancer is one the most prevalent gynecological cancers and, unfortunately, has a poor prognosis due to low response rates to traditional treatments. However, the progress in molecular biology and understanding the genetic mechanisms involved in tumor processes offers valuable information that has led to the current classification that describes four molecular subtypes of endometrial cancer. This review focuses on the molecular mechanisms involved in the pathogenesis of endometrial cancers, such as genetic mutations, defects in the DNA mismatch repair pathway, epigenetic changes, or dysregulation in angiogenic or hormonal signaling pathways. The preclinical genomic and molecular investigations presented allowed for the identification of some molecules that could be used as biomarkers to diagnose, predict, and monitor the progression of endometrial cancer. Besides the therapies known in clinical practice, targeted therapy is described as a new cancer treatment that involves identifying specific molecular targets in tumor cells. By selectively inhibiting these targets, key signaling pathways involved in cancer progression can be disrupted while normal cells are protected. The connection between molecular biomarkers and targeted therapy is vital in the fight against cancer. Ongoing research and clinical trials are exploring the use of standard therapy agents in combination with other treatment strategies like immunotherapy and anti-angiogenesis therapy to improve outcomes and personalize treatment for patients with endometrial cancer. This approach has the potential to transform the management of cancer patients. In conclusion, enhancing molecular tools is essential for stratifying the risk and guiding surgery, adjuvant therapy, and cancer treatment for women with endometrial cancer. In addition, the information from this review may have an essential value in the personalized therapy approach for endometrial cancer to improve the patient’s life.

## 1. Introduction

Endometrial cancer (EC) has its origin in the endometrium, which is the mucous membrane lining the uterus. It is one of the most frequent gynecologic cancers, and it predominantly affects postmenopausal women, although it can also be found in younger women (under 40 years of age) or in premenopausal women. EC usually presents with abnormal uterine bleeding, such as postmenopausal or irregular menstrual bleeding [1]. The etiology and pathology of EC are influenced by a combination of genetic, hormonal, and environmental factors [2]. According to the latest available data from the World Health Organization’s International Agency for Research on Cancer (IARC) and the European Cancer Information System (ECIS), the incidence of EC in Europe has been increasing over the past few decades, partly due to changes in obesity prevalence, hormonal factors, and improvements in cancer detection and diagnosis [3,4]. Mortality rates tend to be lower than incidence rates, reflecting improvements in treatment and outcomes for EC patients, particularly those diagnosed at early stages [5]. EC is a heterogeneous disease with multiple risk factors, histological subtypes, and treatment options. Early detection and complex treatment approaches are essential in optimizing results and improving survival rates for EC. The diagnosis is based on clinical evaluation, imaging techniques (such as transvaginal sonography or MRI), and histopathological examination, which implies prior tissue sampling (usually an endometrial biopsy/curettage) [6,7,8].

Menarche age, age at the last birth, breastfeeding, fertility treatments, and parity can represent a series of factors involved in the appearance of EC. Also, the use of nonsteroidal anti-inflammatory drugs, oral contraceptives, and IUDs or the existence of some medical conditions such as polycystic ovarian syndrome, metabolic syndrome, systemic lupus erythematosus, diabetes mellitus, hypertension, and obesity can lead to the occurrence and advancement of EC [9,10,11]. Unlike the aforementioned pathologies, the percentage of malignant pathologies of the endometrium and their impact on fertility are still unknown due to the lack of reports based on large groups of patients [12]. EC diagnosed in women under 40 years old is usually a very well-differentiated focal endometrioid tumor, restricted in the endometrium or with minimal myometrial invasion, with a high expression of estrogen and progesterone receptors. Clinical observation shows that 8% of patients under 40 years of age with EC are diagnosed with stage I (50–90%) [13,14].

Classification and staging of EC are important for guiding treatment decisions and generating the most realistic prognosis. The traditional histologic classification elaborated by Bokhman divides EC into estrogen-dependent carcinomas (EEC), which include the following subtypes: endometrioid adenocarcinoma and mucinous adenocarcinoma or type I, and non-endometrioid endometrial carcinomas (NEEC) or less frequent tumors (<20%), which encompass more aggressive subtypes, such as serous carcinoma, clear cell carcinoma, and carcinosarcoma (also known as malignant mixed Müllerian tumor or MMMT) [15].

The evolution of genetic and molecular studies has offered valuable information that has led to the current classification that describes four molecular subtypes of EC. The Cancer Genome Atlas (TCGA) has played a significant role in advancing our understanding of the molecular landscape of EC [16]. The TCGA described in 2022 four molecular subtypes of EC based on genomic characteristics: (1) POLE-mutated (ultra mutated, ≥100 mutations/Mb) tumors are characterized by mutations in the DNA polymerase epsilon (POLE) gene; these mutations lead to distinct characteristics such as high mutational burden, microsatellite stability, significant immune cell infiltration, and generally favorable prognosis [17], and they are often well-differentiated endometrioid carcinomas. (2) Microsatellite instability (MSI-H) tumors are characterized by defects in the DNA mismatch repair (MMR) system and when the errors were accumulated in microsatellite regions of the genome, leading to MSI; often, MSI-H is accompanied by a high mutational burden, and significant immune response due the infiltration of immune cells, into the tumor microenvironment. All of these factors influence the behavior, prognosis, and treatment response of these tumors. Because this subtype appears at an earlier stage, it is associated with a better prognosis and is more commonly found in endometrioid carcinomas [18,19]. This subtype is associated with a better prognosis and is more commonly found in endometrioid carcinomas. (3) Copy number low (CNL)/microsatellite stable (MSS) tumors have a relatively stable genome with fewer genetic alterations and lower mutation rates [20]. This subtype is enriched for endometrioid carcinomas and is linked with a moderately favorable prognosis. (4) Copy number high (CNH) tumors have a high level of chromosomal instability and numerous copy number alterations, especially in TP53 and PIK3CA; this subtype is enriched for serous carcinomas that are more aggressive and is associated with a poorer prognosis [21].

In this comprehensive review, we have thoroughly focused in the first part on the various molecular mechanisms responsible for the development and progression of ECs. This information helps to identify biomarkers that enhance the understanding of EC pathology and guide the development of effective treatments. So, in the second part of the review, we present the known diagnostic or prognostic biomarkers or those in different stages of research in the laboratory or in clinical trials. At the end of the article, some molecules are presented that can be considered molecular targets and allow the approach of targeted therapy strategies for endometrial cancer.

## 2. Molecular Mechanisms Involved in the Pathogenesis of Endometrial Cancers

**Figure 1:**
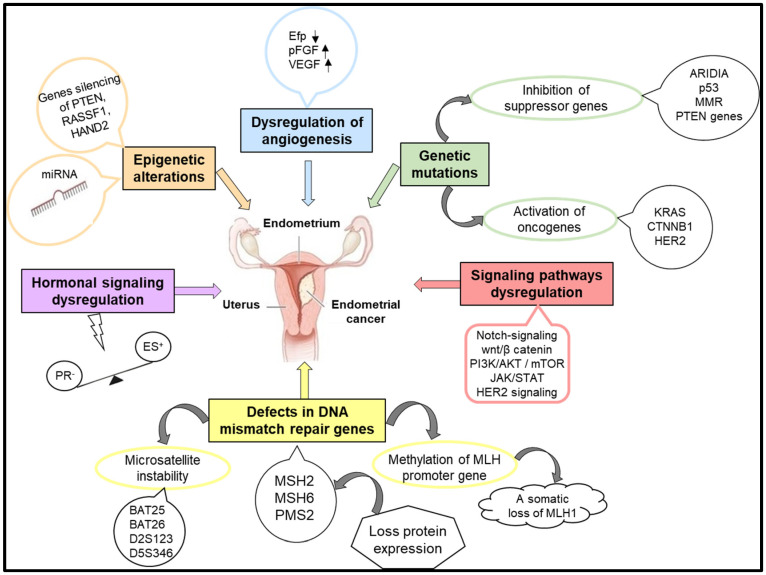
The molecular mechanisms involved in the occurrence and evolution of endometrial cancer.

The pathogenesis of endometrial cancer implies complex molecular mechanisms responsible for the appearance of genetic mutations, the activation of oncogenes accompanied by the inactivation of tumor suppressor genes, defects in the DNA mismatch repair (MMR) pathway, imbalance in hormonal signaling pathways, epigenetic modifications, or disorders in angiogenesis (Figure 1) [22,23].

### 2.1. Genetic Mutations

Genetic mutations are essential factors in the development and progression of EC. These mutations can affect a series of genes involved in different cellular processes, such as the regulation of cellular cycle, DNA repair, apoptosis, and hormonal signaling. One of the most frequent genetic modifications in EC is that found on *PTEN* suppressor gene (phosphatase and tensin homolog), which regulates cell growth, survival, and proliferation. Many endometrial tumors exhibit a loss of PTEN function that can occur due to mutations, deletions, or epigenetic silencing. This loss of PTEN function leads to the dysregulation of the PI3K/AKT/mTOR pathway, which promotes abnormal cell growth and proliferation. This, in turn, contributes to the development and progression of EC [24]. PTEN status in EC patients can have prognostic and therapeutic implications. Tumors with PTEN alterations may exhibit more aggressive behavior and resistance to certain therapies. Additionally, targeting the PI3K/AKT/mTOR pathway, either directly or indirectly, represents a potential therapeutic strategy for ECs with PTEN mutations or loss of PTEN function. Understanding the role of PTEN in endometrial cancers is essential for developing targeted therapies and improving patient outcomes [25,26].

Furthermore, mutations in the *TP53* gene determine the loss of its suppressive functions, allowing cells to avoid apoptosis and to accumulate genetic modifications. Thus, the presence of TP53 mutations in ECs is associated with high aggressiveness and a poor prognosis [27].

In endometrial cancers, especially in clear cell and endometrioid subtypes, mutations of ARID1A (AT-rich interaction domain 1A gene), which codes a subunit of the SWI/SNF chromatin remodeling complex, are very frequent. The presence of ARID1A mutations induces disorders in the process of chromatin remodeling and that of genetic expression, contributing to tumorigenesis and the progression of the tumor [28]. In high-grade endometrioid carcinomas, ARID1A mutations are present in over 40% of cases, while in low-grade endometrioid carcinomas, they are observed in only 25% of cases [29]. ARID1A interacts with numerous transcription factors and other signaling molecules, so the recorded disturbances induce changes at the level of several molecular pathways. Some studies have shown that changes in the ARID1A gene in EC influence PI3K and protein kinase B (AKT) signaling pathways [30,31]. Also, an association was found between ARID1A loss with microsatellite instability (MSI) and mismatch repair (MMR) deficiency in the case of uterine endometrioid carcinoma. In clear cell carcinoma, loss of ARID1A is associated with aberrant expression of the p53 protein or mutations of PTEN and PIK3CA [32]. In ARID1A-deficient endometrial cancer cells, the level of FOXO1 increased while the mitogen-activated protein kinase pathway (MAPK) and insulin-like growth factor-1 signaling pathway decreased [33]. In endometrial carcinogenesis, dysregulation of the ARID1A gene can influence several genes such as the skeletal phosphatase 2 subunit alpha gene (PPP2R1A), PTEN, PIK3CA, KRAS, catenin beta 1 gene (CTNNB1), TP53, proto-oncogene B-Raf, serine/threonine kinase gene (BRAF), and PPP2R5C. Thus, starting from the level of dysregulation of ARID1A, a classification was made based on the mutational profile of endometrial carcinomas [34]. The mechanism of action of ARID1A in the initiation and progression of cancer cells needs to be clarified in order to develop new diagnostic tools and to design new, more effective therapeutic approaches.

Inactivation of the PTEN tumor suppressor gene is associated with the inactivation of other tumor suppressor genes, such as TP53 (p53) and ARID1A, in EC [35]. Loss of function of these genes removes important regulatory mechanisms that normally inhibit tumor growth and promote apoptosis [36]. Oncogenes, such as KRAS, CTNNB1 (β-catenin), and HER2/neu (human epidermal growth factor receptor 2), when mutated or activated, induce the stimulation of proliferation and extended survival of tumor cells, promoting the invasion and metastases of EC [37]. KRAS is a proto-oncogene involved in transmitting signals from the cell-surface receptors to the cell’s nucleus. KRAS mutations have been observed especially in the endometrioid subtype and are responsible for the activation of its MAPK/ERK signaling pathway, which contributes to the growth and progression of the tumor [38]. CTNNB1 codes β-catenin, which is involved in cellular adherence and in the regulation of the Wnt signaling pathway [39]. Mutations in CTNNB1 lead to the stabilization and accumulation of β-catenin in the cytoplasm, followed by its translocation in the nucleus, where it acts as a transcriptional co-activator, promoting the expression of genes, such as c-Myc, cyclin D1, surviving, and MMP7 (matrix metalloproteinase 7). Increased expression of these genes promotes cell cycle progression, inhibits apoptosis, and enhances cellular proliferation, contributing to tumor growth and progression [40,41].

HER2/neu is a tyrosine kinase receptor in the EGFR family (epidermal growth factor receptor). Amplification or overexpression of HER2/neu leads to the constitutive activation of its signaling pathways, PI3K/AKT/mTOR and MAPK/ERK, which promote proliferation and cell survival [42]. Targeting these oncogenic pathways represents a potential therapeutic strategy for subsets of EC patients with specific oncogene mutations [43].

### 2.2. Defects in DNA Mismatch Repair Genes

Defects in DNA mismatch repair genes, such as MLH1, MSH2, MSH6, and PMS2, are associated with microsatellite instability-high (MSI-H) endometrial cancers. MMR deficiency leads to the accumulation of DNA replication errors and genomic instability, predisposing cells to cancer development [44]. In some endometrial cancers, sporadic modifications of the MMR pathway can occur, without any inherited genetic predisposition. These modifications are the result of somatic mutations or epigenetic modifications that affect the MMR genes, leading to a dysfunction of MMR, accompanied by a growth in the levels of microsatellites (MSI-H). It is relevant to mention the significantly high risk of developing endometrial cancer in women with Lynch syndrome, which is an inherited disease caused by mutations of MMR genes (especially MLH1, MSH2, MSH6, and PMS2) [45]. Testing of the expression of MSI or MMR protein in tumoral tissue with the help of immunohistochemistry techniques is used to identify endometrial cancer patients with an MMR deficit. Identifying the MMR status can be useful in guiding therapeutic decisions because patients with endometrial cancer, and an MMR deficit can have a different response to treatment [46].

### 2.3. Hormonal Signaling

Hormonal signaling is an essential mechanism involved in the development and progression of EC. The endometrium is very sensitive to hormonal fluctuations, especially those of estrogen and progesterone. Imbalances in estrogen and progesterone levels, as well as alterations in hormone receptor expression, contribute to the pathogenesis of endometrial cancer [47].

The growth of estrogen levels (ES) due to endogen factors (such as obesity, polycystic ovarian syndrome, or estrogen-secreting tumors) or exogen factors (such as hormonal substitution therapy without any progestin) can lead to excessive growth of endometrial cells and can increase the risk for developing endometrial cancer [48]. ES exerts its effects through binding with estrogen specific receptors, ER-alfa and ER-beta, thus activating the signaling pathways responsible for cellular proliferation, angiogenesis stimulation, and apoptosis inhibition. A high expression of ER-alfa or the loss of ER-beta expression can contribute to the development of EC [49]. Furthermore, the disturbances that can occur in the metabolism of estrogen inside the body (hydroxylation, methylation) can lead to the formation of genotoxic metabolites or the accumulation of estrogenic compound (2-hydroxiestrone (2-OHEI) and 16α-hydroxy estrone (16α-OHEI)). An increased ratio between 16α-OHEI and 2-OHEI has been associated with a high risk of endometrial cancer [50,51]. Progesterone (PR), another ovarian hormone, acts to counterbalance the effects of estrogen on the endometrium. PR has anti-proliferative effects on the endometrial cells by inhibiting the progression of the cellular cycle and DNA synthesis [52]. Progesterone induces a decrease in the expression of the genes involved in the progression of the cellular cycle, such as cyclins and cyclin-dependent kinases (CDK), and it also regulates the expression of cellular cycle inhibitors, such as p21 and p27, and, thus, it inhibits cellular proliferation. A deficiency in progesterone signaling, which can occur due to the absence of ovulation or due to an inadequate luteal phase, can lead to estrogen stimulation and increase the risk for EC [53,54]. A dysregulation in hormonal signaling can induce abnormal proliferation, genomic instability, and angiogenesis inside the endometrium, thus predisposing to the development of EC [55]. Understanding the interactions between hormonal factors and the molecular pathways involved in the pathogenesis of EC is crucial to find new molecular targets, which can lead to new preventive or therapeutic strategies [56].

### 2.4. Epigenetic Modifications

Epigenetic modifications occur in EC, involving DNA methylation, histone modification, and micro-RNA (miRNA) dysregulation, leading to aberrant gene expressions [57]. An epigenetic modification that can appear in endometrial cancer is the aberrant methylation of DNA at the level of CpG islets, which is induced by the activation of DNA-methyltransferases (DNMT), and it determines gene expression modifications by inhibiting or activating transcription [58]. Also, DNA methylation involves the hypermethylation of the promotor regions of tumor suppressor genes, which can lead to their silencing, or the hypomethylation of oncogenes, which can lead to their overexpression, thus promoting tumorigenesis [59].

Modifications that can appear in histones include the acetylation, methylation, and phosphorylation processes, and they affect the structure of chromatin and the transcription of genes, contributing to the progression of EC [60,61]. The process of histone methylation can take place on lysine residues (mono-, di-, or tri-methylation) or arginine residues (mono- or di-methylation). This process is regulated by histone methyl-transferases (HMT) and histone demethylases (HMT). The abnormal modification of histones affects gene expression [62]. Histone acetylation is dependent on the balance between histone acetyltransferases (HAT) and histone deacetylases (HDAC), which influence access to transcription factors and thus influence gene expression [63]. Furthermore, histone acetylation/deacetylation influences the gene expression of non-histone proteins, which are involved in tumor progression and metastases. Thus, an increased level of HDAC (HDAC1, HDAC2, and HDAC3) found in EC is linked to poor prognosis [64].

### 2.5. Angiogenesis

Angiogenesis is a physiological process that ensures the formation of new blood vessels, and it contributes to wound healing and embryological development. In EC, the dysregulation of angiogenic signaling pathways leads to the abnormal formations of new blood vessels and so it contributes to tumor growth, invasion, and metastasis by providing oxygen and nutrients for tumor cells [65]. A series of factors contribute to angiogenic signaling in endometrial cancer. Inside the tumor, the proliferative capacity of tumor cells exceeds the process of blood vessel growth, and it determines the appearance of areas characterized by a lack of oxygen (hypoxia). Hypoxia-inducible factor-1α (HIF-1α) is a key transcription factor that regulates cellular response to hypoxia [66]. Hypoxia triggers the increased release of pro-angiogenic factors, such as vascular endothelial growth factor (VEGF) [67], fibroblast growth factor (FGF) [68], and platelet-derived growth factor (PDGF) [69]. Angiopoietins (Ang) and their receptor Tie2 play a crucial role in regulating the stability and remodeling of blood vessels. Overexpression of angiopoietins and the activation of Tie2 can promote abnormal angiogenesis in endometrial cancer [70].

Endometrial tumor cells secrete anti-angiogenic factors in small quantities, factors such as thrombospondin-1 and endostatin, which have a role in inhibiting angiogenesis. This imbalance between pro-angiogenic factors and anti-angiogenic factors promotes angiogenesis inside the tumor microenvironment. Dysregulation of the Notch signaling pathway is associated with the blood vessel maturation process and is involved in promoting angiogenesis and tumor progression in EC [71].

Another signaling pathway that is frequently deregulated in endometrial cancer is represented by the PI3K/AKT/mTOR pathway. The activation of this pathway can promote angiogenesis by positive regulation of the expression of VEGF, increasing endothelial cell proliferation and tumoral cell survival [72].

Also, epigenetic alterations, such as DNA methylation [73] and histone modification [74], can impact the expression of genes involved in angiogenesis and the formation of abnormal blood vessels in tumors. Furthermore, matrix metalloproteinases (MMP) facilitate tumoral angiogenesis by degrading the extracellular matrix, allowing endothelial cells to migrate and form new blood vessels [41].

In post-transcription regulation of gene expression in EC, a major role is played by micro-RNA (miARN). These are short, non-coding, single-stranded RNA molecules that regulate post-transcription gene expression through binding with mRNA [75]. miRNAs modulate processes such as cellular division, proliferation, differentiation, apoptosis, and blood vessel formation [76]. The modified expression of miRNAs has also been found in endometrial cancer, and it can influence angiogenesis processes by targeting genes involved in the functionality of endothelial cells, vascular permeability, and angiogenic signaling [77,78].

In conclusion, epigenetic modifications and the dysregulation of angiogenesis have an important role in the development and progression of EC. Deciphering and understanding these processes can lead to identifying new molecular biomarkers or therapeutic targets useful in finding more efficient treatments for EC.

## 3. Biomarkers in Endometrial Cancer

Molecular biomarkers are molecules that indicate a biological or pathological process or therapy response. They can be found in tissues, blood, urine, or other bodily fluids and analyzed using various molecular techniques, such as polymerase chain reaction (PCR), fluorescence in situ hybridization (FISH), immunohistochemistry (IHC), and next-generation sequencing (NGS).

**Figure 2:**
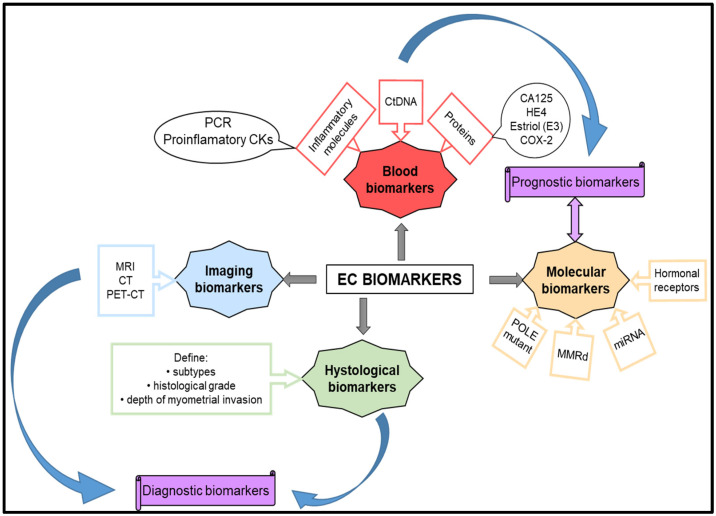
Biomarkers for diagnosis and prognostic endometrial cancer.

Molecular biomarkers are essential in diagnosing, prognosis, predicting therapeutic response, and monitoring disease progression in endometrial cancer (Figure 2). Continuous advancements in molecular techniques and genomic profiling technologies are expanding the range of molecular biomarkers available for use in the management of endometrial cancer.

Examining the endometrial tissue obtained through biopsy or surgical resection is still the most reliable method for diagnosing EC. Pathologists rely on histological features, such as glandular architecture, nuclear morphology, and mitotic activity to differentiate between benign conditions, hyperplasia, and malignancy [79].

Imaging biomarkers are a crucial factor in assessing the response of EC to treatment. Two imaging techniques, namely, magnetic resonance imaging (MRI) and computed tomography (CT) scans, help monitor changes in tumor size, volume, and enhancement patterns revealed by imaging studies, indicating response or progression during or after treatment [80,81]. Positron emission tomography-computed tomography (PET-CT) scans, on the other hand, use radioactive tracers to detect changes in tumor metabolic activity over time, thus being particularly useful for evaluating treatment response in advanced or recurrent EC [82].

Serum biomarkers facilitate early detection, precise diagnosis, prognostic evaluation, and treatment response monitoring. Clinical evaluation of serum biomarkers alongside imaging and clinical data can ensure an accurate diagnosis and the selection of an optimal treatment option [83]. HE4 (human epididymis protein 4), together with other serum biomarkers, such as CA-125, CA724, CA19-9 [84,85,86], and YKL-40, also known as chitinase-3-like 1 protein (CHI3L1) and DKK-3 [87], can contribute to the detection and monitoring of endometrial cancer. High levels of HE4 have been associated with advanced-stage disease and can indicate the aggressiveness of the tumor. Thus, high serum levels of HE4 are associated with advanced stage disease, as well as with the implication of the endocervical stroma and deep myometrial invasion [88]. A serum HE4 value higher than 201.3 pmol/L indicates a high risk for recurrence for endometrioid cancers [89,90]. In conclusion, monitoring the modifications of HE4 levels can, in time, offer additional information about disease progression and the risk of recurrence.

A series of serum proteins are being studied as potential biomarkers for EC, but the so far obtained results have not been enough to be validated in clinical practice. Such a protein is Dickkopf-1 (DKK-1), which is involved in embryological development and has been found in high concentrations in the serum of patients with advanced-stage EC (stages III–IV) [91]. Also, there are studies that have identified high serum levels of DJ-1 protein (part of the peptidase C56 family) in patients with EC, especially in non-endometrioid subtypes [92,93]. Other protein biomarkers such as osteopontin, leptin, and tumor-associated glycoprotein 72 (TAG-72) are under study. Osteopontin is a glycoprotein found in increased quantity in different cancers, including EC. Its expression is linked to the advancement of the tumor, metastasis, and unfavorable results in EC patients. It assists in promoting tumor cell survival, invasion, and angiogenesis by interacting with cell surface receptors and signaling pathways involved in these processes. Furthermore, osteopontin can impact the immune response in the tumor microenvironment, influencing the anti-tumor immune response and immune evasion mechanisms. The osteopontin levels in serum or tumor tissue have been studied as a possible biomarker for predicting disease severity, treatment response, and patient outcomes in endometrial cancer [94]. Leptin is a hormone mainly produced by adipose tissue. It has pro-inflammatory and pro-angiogenic properties and is linked to cancer development and progression, including EC [95]. Leptin receptors are present in endometrial cancer cells, and leptin signaling pathways can promote the proliferation, migration, and invasion of tumor cells. High levels of circulating leptin are linked to an increased risk of EC, particularly in postmenopausal women and obese individuals [96].

Tumor-associated glycoprotein 72 (TAG-72) is a glycoprotein present on the surface of cells that is often found in higher levels in EC and other types of cancers. TAG-72 can be detected in tumor tissue or serum and has been studied as a potential biomarker to help diagnose and track cancer progression. In endometrial cancer patients, TAG-72 expression has been linked to more advanced stages of disease, lymph node metastases, and poorer prognosis [97].

Hormonal biomarkers such as estrogen and progesterone receptors and biomarkers associated with inflammation and immune response such as C-reactive protein (CRP) and some cytokines can be detected in the serum of patients with EC [98]. These biomarkers can provide information regarding tumor–host interactions and immune response, and they may hold prognostic significance [99].

Biomarkers associated with angiogenesis, such as VEGF and its soluble receptors (RVEGF), can be measured in the serum of patients with EC [100]. These biomarkers are indicative of tumor angiogenesis and can provide insights into disease aggressiveness and the efficacy of anti-angiogenic therapies.

Tumor cells release fragments of tumor-derived DNA into the bloodstream, known as circulating tumor DNA (ctDNA). Analysis of ctDNA can be performed using various techniques, such as digital PCR (dPCR), next-generation sequencing (NGS), and allele-specific PCR (AS-PCR). These techniques allow for detecting and quantifying specific mutations or genetic changes in ctDNA. By analyzing ctDNA, we can obtain information about the genetic mutations, changes in the number of copies, and methylation patterns of the tumor [101]. These data are helpful for early detection, monitoring the response to treatment, and detecting minimal residual disease or recurrence in EC patients [102].

### Prognostic Biomarkers Are Indicators That Predict the Course or Outcome of a Disease

They aid clinicians in identifying the risk of recurrence or overall survival, regardless of the treatment. In EC, numerous biomarkers have been discovered as prognostic indicators. Histopathological examination of tissue or biopsy samples is used to identify biomarkers that aid in the diagnosis and prognosis of endometrial cancers [103]. Histological grade is a strong prognostic factor in EC and is determined by the tumor’s degree of cellular differentiation and architectural features. Tumors are typically categorized as Grade 1 (well-differentiated), Grade 2 (moderately differentiated), or Grade 3 (poorly differentiated). Higher-grade tumors are associated with a worse prognosis and an increased risk of recurrence. The stage of a tumor describes how far it has spread and is a critical factor in predicting the outcome of endometrial cancer. The International Federation of Gynecology and Obstetrics (FIGO) staging system determines the tumor stage based on various factors, such as tumor size, depth of invasion, involvement of nearby structures, and the presence of metastases. Patients with advanced-stage tumors (FIGO stage III or IV) generally have worse outcomes than those with early stage disease [104]. Lymphovascular invasion (LVI) is a significant prognostic factor in EC. It refers to the presence of tumor cells within lymphatic or blood vessels. LVI indicates a higher risk of lymph node metastasis and distant recurrence, which leads to a poorer prognosis [105]. Tumor size is another important prognostic factor in EC. Tumors with diameters greater than 2 cm indicate a worse prognosis [106]. The depth of myometrial invasion, which is determined by the extent of tumor infiltration into the uterine wall, is also an important prognostic factor. Deep myometrial invasion, defined as greater than 50% of myometrial thickness, is associated with a higher risk of lymph node involvement and distant metastasis, leading to a poorer prognosis [107].

Immunohistochemical staining for p53 protein expression or molecular analysis to test *TP53* gene mutations can assess p53 status. Alterations in TP53, which encodes the p53 protein, are associated with high-grade tumors, aggressive tumor behavior, and poorer prognosis in EC [108]. Ki-67 expression is another marker of cellular proliferation, and high levels of its expression are associated with aggressive tumor behavior and a worse prognosis in EC [109].

Immunohistochemical staining can be used to analyze the expression of MMR proteins (such as MLH1, MSH2, MSH6, and PMS2), which can help define deficient DNA mismatch repair (dMMR). These proteins are associated with a better prognosis and improved response to immunotherapy in EC [110,111].

MicroRNAs (miRNAs) are small non-coding RNAs that play important roles in the regulation of gene expression. Dysregulation of miRNA expression has been implicated in the pathogenesis, progression, and prognosis of various cancers, including endometrial cancer. Several studies have investigated the potential of miRNAs as molecular biomarkers for prognosis in EC [112]. Overexpression of miR-21 and miR-183-5p have been associated with tumor aggressiveness, lymph node metastasis, and poorer prognosis in endometrial cancer [77]. Reduced expression of miR-205 has been associated with advanced-stage disease and poorer prognosis in endometrial cancer. Low levels of miR-192, miR-194, miR-215, miR-99a, and miR-100 expression has been correlated with tumor aggressiveness and metastasis in endometrial cancer [113]. Dysregulation of the miR-200 family members (miR-200a, miR-200b, miR-200c, miR-141, miR-429) has been associated with epithelial-to-mesenchymal transition (EMT), tumor aggressiveness, and metastasis in EC [114]. The miR-34 family members are known tumor suppressor miRNAs, and the decrease in their expression has been associated with tumor aggressiveness, high-grade tumors, and poorer prognosis in EC [115]. These miRNAs have shown promise as potential prognostic biomarkers. However, further validation in larger, well-designed studies is needed to confirm their clinical utility and to establish standardized protocols for their assessment in routine clinical practice.

Tumor-infiltrating lymphocytes (TILs) are immune cells that have migrated into the tumor microenvironment and can provide information about the tumor’s biology and the patient’s immune response [116]. In patients with endometrial tumors, a higher density of TILs is correlated with a better prognosis, including more prolonged overall survival and progression-free survival. TILs, especially cytotoxic CD8+ T cells, are indicative of a robust anti-tumor immune response, which can help control tumor growth and spread. Thus, TILs can be considered prognostic biomarkers in endometrial cancers, but their prognostic value varies among the different molecular subtypes of endometrial cancer. POLE ultramutated and MSI-H subtypes, which are associated with high mutation rates and increased neoantigen load, tend to have higher levels of TILs and generally better outcomes. On the contrary, the copy number high (CN-H) subtype, which is associated with more aggressive disease, often shows lower levels of TILs and poorer prognosis [117]. Also, assessing TILs in endometrial cancer can help identify patients who might benefit from immunotherapy, making them a valuable tool for personalized treatment approaches [118]. Combining TIL assessment with other biomarkers, such as PD-L1 expression, mutational load, and molecular subtyping, can enhance the prognostic and predictive power of endometrial cancer management.

The prognostic biomarkers help clinicians stratify patients into different risk groups and guide treatment decisions, such as the intensity of adjuvant therapy and the frequency of surveillance. Integrating prognostic biomarkers into clinical practice enables personalized management of endometrial cancer patients based on their individual risk factors.

## 4. Endometrial Cancer Therapy between Standard Approaches and Future Directions

### 4.1. Standard Therapy

Therapeutic approaches for treating endometrial cancer depend on various factors, such as the stage and grade of the tumor, the depth of myometrial invasion, the status of lymphovascular space involvement, the presence of specific biomarkers, and the patient’s overall health. Management of endometrial cancer at diagnosis involves surgical methods. Thus, minimally invasive surgery, such as laparoscopic or robot-assisted surgery, can be used for early stage endometrial cancer, which can lead to good results with less morbidity and a shorter postoperative recovery period [119]. Patients with high-risk features, such as deep myometrial invasion, high-grade tumors, or lymphovascular invasion, may be recommended to undergo postoperative radiation therapy. External beam radiation therapy or brachytherapy can be used to kill cancer cells [120]. Radiation therapy can be used as neoadjuvant therapy to shrink the tumor before surgery or as adjuvant therapy to kill any remaining cancer cells after surgery [121].

In the case when the tumors cannot undergo surgery, chemotherapy may be used as the primary treatment to kill cancer cells. Often, chemotherapy may be applied after surgery to destroy any remaining cancer cells. Chemotherapy is a standard treatment for endometrial cancer. The chemotherapy drugs used for this treatment are Paclitaxel, Carboplatin or Cisplatin, Doxorubicin (Adriamycin), Epirubicin, and Ifosfamide [122]. These drugs belong to different categories: taxane, platinum-based, anthracycline, and alkylating agent chemotherapy drugs. These chemotherapy drugs interfere with the growth and division of cancer cells by damaging the DNA of cancer cells and may be used alone or in various combinations [123,124]. The dosage and specific regimen of these chemotherapeutic drugs depend on the stage and characteristics of the endometrial cancer. A multidisciplinary team of healthcare professionals determines the appropriate combination of medications for a particular patient [125].

Hormonal therapy is a treatment option that has been used for a long time to treat recurrent endometrial cancer. It is particularly useful for patients with slow-growing, low-grade tumors or for patients for whom other treatments may be too toxic. Hormonal therapy can be used for women with estrogen-receptor-positive advanced or recurrent endometrial cancer. It involves using medications to block the estrogen effects on cancer cells.

Although progestins and megestrol acetate therapy are FDA-approved for treating advanced and recurrent endometrial cancer patients, recent studies have presented contradictory results [126,127]. Thus, some studies have highlighted a correlation between the expression level of ER and PR receptors in primary tumors compared to recurrent tumors. In contrast, other studies deny this correlation [128,129]. These findings have opened new directions for hormone therapy. Studies in molecular biology have revealed that the MAPK pathway is involved in ER alpha signaling and activates other molecules such as ERK and AKT [130,131]. In the case of PR signaling, the PI3K/AKT/mTOR pathway is also activated in addition to the MAPK pathway. All this led to the approach of some studies that combine endocrine therapy with mTOR inhibitor. Endometrial cancer preclinical studies have demonstrated that inhibiting the PI3K/Akt pathway can reverse progestin resistance in EC models. This approach has been pursued in clinical trials [131].

Luteinizing-hormone-releasing hormone agonists (LHRH agonists) lower estrogen levels in women who are premenopausal and could be used in the therapy of EC [132]. It has been found that the use of aromatase inhibitors can be effective in treating endometrioid tumors that have a positive estrogen receptor status. Aromatase is an enzyme that is responsible for estrogen biosynthesis and has been found in malignant endometrial tissue. Aromatase inhibitors that can regulate estrogen levels and exert antitumor effects in endometrial carcinoma by blocking aromatase have been developed. Studies have shown that aromatase inhibitors such as letrozole or anastrozole can be used to treat EC, particularly in postmenopausal women with hormone-receptor-positive tumors [133]. These drugs are still being studied for how to use them best to treat EC.

### 4.2. Targeted Therapy Strategies in Endometrial Cancer

Targeted therapy refers to a specific type of cancer treatment that involves identifying specific molecular targets in cancer cells responsible for tumor formation and growth. These molecular targets are then selectively inhibited or modulated by targeted therapies, which disrupt key signaling pathways involved in cancer progression while protecting normal cells. This link between molecular markers and targeted therapy is crucial in the fight against cancer.

Targeted therapy is a recent approach to treating EC, and only a few validated drugs are currently being used. These drugs are primarily used to treat high-risk endometrial cancers that have metastasized or relapsed after previous treatment. Some of the therapeutic strategies presented are still part of a preclinical or clinical study. The molecular profiling of endometrial tumors is essential for identifying potential biomarkers and guiding targeted therapy decisions [134].

#### 4.2.1. Targeting the PI3K/AKT/mTOR Signaling Pathway

The PI3K/AKT/mTOR signaling pathway is often disrupted in EC, making it a target for therapeutic intervention. PI3K/AKT/mTOR inhibitors are a class of targeted therapies that act by inhibiting various components of the PI3K/AKT/mTOR signaling pathway. PI3K inhibitors, such as Alpelisib, Taselisib, and Buparlisib, block the activity of the PI3K enzyme (phosphoinositide 3-kinase) that is responsible for converting phosphatidylinositol-4,5-bisphosphate (PIP2) to phosphatidylinositol-3,4,5-trisphosphate (PIP3), which in turn activates the downstream AKT and mTOR (mammalian target of rapamycin) signaling pathways [135]. Capivasertib and Ipatasertib act as AKT inhibitors to block the activation of AKT (protein kinase B), thereby preventing the phosphorylation and inactivation of pro-apoptotic proteins and promoting cell cycle arrest and apoptosis in cancer cells. Everolimus and temsirolimus are used as mTOR inhibitors. They block the activity of mTOR complex 1 (mTORC1), leading to decreased protein synthesis, cell cycle arrest, and autophagy in cancer cells. Tumors with PIK3CA mutations or other PI3K pathway alterations are more likely to respond to PI3K/AKT/mTOR inhibitors. Everolimus is a medication approved for clinical use in treating advanced or recurrent EC. It can be used alone or in combination with hormonal therapy like exemestane. This treatment is specifically designed for selected EC patients who have hormone-receptor-positive, HER2-negative disease [136].

PI3K inhibitors like Alpelisib, Taselisib, Buparlisib, and an AKT inhibitor known as Capivasertib are being evaluated in clinical trials as single agents or in combination with other targeted therapies or chemotherapy. The effectiveness of using single-agent PI3K inhibitors for the treatment of EC has been limited due to drug resistance and toxicity. However, combining PI3K inhibitors with standard chemotherapy or radiation therapy may improve the therapeutic response and lead to better outcomes, especially in cases of aggressive or advanced-stage disease [137]. Ongoing clinical trials are currently evaluating the effectiveness, safety, and optimal combinations of PI3K/AKT/mTOR inhibitors in different settings of EC to enhance treatment outcomes and patient care [138,139]. Alpelisib is currently undergoing clinical trials as a standalone treatment or in combination with hormonal therapy or other targeted therapies to effectively treat advanced or recurrent EC. This drug has shown exceptional results in treating tumors with PIK3CA mutations or other alterations in the PI3K pathway [140]. Also, the combination of PI3K/AKT inhibitors with hormonal therapies, such as progesterone or aromatase inhibitors (such as exemestane), may synergistically inhibit tumor growth and enhance response rates in hormone-receptor-positive endometrial cancers [47,141,142].

Combining PI3K/AKT/mTOR inhibitors with immune checkpoint inhibitors like Pembrolizumab or Nivolumab offers a promising approach regarding the improvement of antitumor immune response and enhancement of therapeutic outcomes in EC. This strategy is particularly effective for tumors with microsatellite instability-high (MSI-H) or deficient mismatch repair (dMMR) [143,144].

#### 4.2.2. Targeting Angiogenesis

Angiogenesis inhibitors are a type of medication that hinders the growth of new blood vessels and is responsible for supplying cancer cells with nutrients. Antiangiogenic agents can target the vascular endothelial growth factor (VEGF). VEGF plays a crucial role in angiogenesis, and Bevacizumab, a monoclonal antibody, blocks its action, thereby preventing the formation of new blood vessels that supply cancer cells with nutrients. Aflibercept (VEGF Trap-Eye) is a fusion protein with a high binding affinity to VEGF-A, VEGF-B, and placental growth factor, thus acting as an angiogenesis inhibitor [145,146]. Thalidomide also has antiangiogenic properties, although its exact mechanism of action is not yet fully understood [147]. There are several drugs that inhibit angiogenesis, and they are currently being tested in clinical trials to determine their effectiveness against the recurrence or metastasis of endometrial cancer. These drugs are called multi-targeted tyrosine kinase inhibitors and include Axitinib, which targets VEGF receptors (VEGFR-1, VEGFR-2, VEGFR-3); Pazopanib and Sunitinib, which target VEGF receptors (VEGFR-1, VEGFR-2, VEGFR-3) and platelet-derived growth factor receptors (PDGFR-α, PDGFR-β); and Nintedanib, which targets VEGF and PDGFR receptors, as well as fibroblast growth factor receptors (FGFR-1, FGFR-2, FGFR-3). One inhibitor of the angiopoietin-Tie2 receptor, which is being tested in endometrial cancer, is Trebananib [148].

Antiangiogenic therapy has emerged as a promising treatment option for endometrial cancer. These targeted therapies can benefit patients with advanced or recurrent EC by blocking angiogenesis, which can disrupt tumor growth and slow down disease progression [149]. Ongoing research and clinical trials are exploring the use of antiangiogenic agents in combination with other treatment strategies to improve outcomes and personalize treatment for patients with EC [150,151,152].

#### 4.2.3. Targeting HER2-Receptors

HER2-positive EC can be treated with HER2-targeted therapy, which has shown great promise. The first and currently the only approved HER2-targeted agent for this indication is trastuzumab, a monoclonal antibody that targets the HER2 receptor. By inhibiting HER2-mediated signaling pathways, trastuzumab leads to decreased proliferation and survival of HER2-positive cancer cells. In clinical practice, trastuzumab (Herceptin) has been approved for selected patients with HER2-positive EC in combination with chemotherapy. For HER2-positive advanced or recurrent EC patients who are not suitable for curative surgery or radiation therapy, this treatment approach is recommended [153,154]. Ongoing research and clinical trials are currently exploring the effectiveness and safety of various HER2-targeted agents, including pertuzumab, lapatinib, and T-DM1. These agents are being studied both alone and in combination with other treatment options to optimize treatment outcomes and create personalized treatment approaches for patients who have HER2-positive EC [155]. It is crucial to identify HER2 overexpression or amplification in endometrial tumors to select appropriate HER2-targeted therapies and to guide personalized treatment decisions in the management of EC [124,156,157].

#### 4.2.4. Immunotherapy

In March 2020, molecular typing of EC was achieved for the first time, and it was included in the guidelines for diagnosis and treatment of EC, which indicates the era of immunotherapy based on tumor microenvironment and genotype. The study of the pathogenesis of EC has revealed the presence of several types of immune system cells in tumor tissue. These cells secrete specific cytokines that modulate the anti-tumor immune response [157]. Immunotherapy is a promising cancer treatment that stimulates the immune system to target tumor cells specifically. Immunotherapy may be used for advanced or recurrent EC with MSI-H or dMMR tumors [158]. Immunotherapy methods such as immune checkpoint inhibitors (ICIs), cancer vaccines, adoptive cell transfer (ACT), lymphocyte-promoting cytokines, and oncolytic viruses are currently being investigated for the treatment of EC [159,160]. One approach involves targeting immune checkpoints, which are molecules that control how the immune system responds to cancer cells. When these checkpoints are overexpressed in cancer cells, they can inhibit the immune system’s ability to identify and attack the tumor [161]. The programmed cell death protein 1 (PD-1) pathway is an important immune checkpoint that has been researched in relation to EC. PD-1 is a protein on the surface of certain immune cells, including T cells. When PD-1 interacts with its ligands, PD-L1 and PD-L2, which are usually present in tumor cells, it can inhibit T cells’ activity, thereby preventing them from attacking the cancer cells. These immune checkpoint signals are considered important targets for immunotherapies that have recently been developed [162,163].

Clinical trials have investigated several immunotherapy drugs designed to target the PD-1 pathway for EC [164,165,166]. In January 2022, Pembrolizumab (Keytruda) showed efficacy in clinical trials and has received FDA (U.S. Food and Drug Administration) approval for specific indications in the treatment of EC. Pembrolizumab (Keytruda) is a checkpoint inhibitor targeting the programmed death receptor-1 (PD-1) protein on T cells, which helps the immune system recognize and attack cancer cells. It is approved for the treatment of advanced or recurrent EC with specific biomarker characteristics, such as microsatellite instability-high (MSI-H) or mismatch repair deficiency (dMMR) that has progressed following prior treatment [167]. In one study report, two cases of recurrent POLE ultra-mutated and MSH6 hyper-mutated endometrial carcinoma refractory to conventional surgical and chemotherapeutic treatments were effectively treated with the anti-PD-1 monoclonal antibody Nivolumab [168].

Numerous other inhibitors are tested in preclinical studies and clinical trials. Aside from Avelumab and Durvalumab, there are promising immune checkpoint inhibitors targeting PD-L1 in EC. Studies of Avelumab effects in patients with recurrent/persistent EC were performed in a trial study (NCT02912572) [169]. The effects of Durvalumab were analyzed in MSI ECs in a phase II PHAE DRA trial (ANZGOG1601) [170].

Another method of immunotherapy involves the use of retroviruses or lentiviruses to modify T cells by introducing chimeric antigen receptors (CARs). Research on CAR T-cell therapy in EC is only carried out in preclinical studies, and the validation of the results, even if they are encouraging, requires further studies [171].

Cancer vaccines are a form of immunotherapy that aims to activate the immune system to recognize and destroy cancer cells. In the case of EC, clinical trials are exploring various cancer vaccines to assess their safety, effectiveness, and potential use in treating the disease. Therapeutic cancer vaccines are composed of tumor/immune cell vaccines; peptide vaccines; and genetic vaccines that consist of DNA, RNA and viral vaccines. There are various peptide-based cancer vaccines that target tumor-associated antigens like NY-ESO-1, MAGE-A, and WT1. These vaccines are currently under clinical trial for treating EC [172,173]. Cancer vaccines are a promising approach for treating EC. Ongoing research and clinical trials are working towards optimizing the design and delivery of these vaccines, identifying predictive biomarkers and developing personalized treatment strategies to improve patient care and outcomes in EC. It is crucial to evaluate cancer vaccines in combination with other immunotherapies, targeted therapies, or standard treatments to explore their potential synergistic effects and develop effective treatment approaches for patients with EC. In order to address the challenges that arise in immunotherapy, it is essential to create personalized treatment strategies to achieve individualized precision treatment. Additionally, it is important to reduce the immunosuppressive environment in patients, particularly the immunosuppressive microenvironment within tumor tissues.

Therapy combinations involving immune checkpoint inhibitors, targeted therapies, chemotherapy, and other immunomodulatory agents are being explored in clinical trials for EC treatment. These approaches aim to improve the antitumor immune response, overcome immunotherapy resistance, and enhance treatment outcomes for patients with advanced or recurrent EC. Ongoing research and clinical trials are crucial to improve outcomes and patient care in EC by optimizing the selection of appropriate combination therapies, identifying predictive biomarkers, and developing personalized treatment strategies (Table 1) [174].

With increased clinical experience and supported by advanced scientific research, tumor immunotherapy can be better understood, leading to an innovative and safe approach to treating cancer patients.

#### 4.2.5. Targeting miRNAs

MicroRNAs (miRNAs) are small, non-coding RNA molecules that play an important role in the regulation of gene expression. Aberrant expression of specific miRNAs has been identified in endometrial cancer, and researchers are currently exploring the potential of using miRNA-based therapies as a treatment approach. There are several approaches to the development and use of miRNA-based therapies. Firstly, synthetic miRNA mimics can be used to restore the expression of tumor-suppressive miRNAs that are downregulated in EC. Secondly, miRNA inhibitors, such as antisense oligonucleotides or antagomirs, can suppress the expression of oncogenic miRNAs that are overexpressed in EC. Lastly, miRNA delivery vehicles, such as lipid nanoparticles or viral vectors, can deliver miRNA mimics or inhibitors to tumor cells in vivo. These delivery systems can enhance the stability and specificity of miRNA-based therapies, improving their efficacy in treating EC [184,185].

Several miRNAs, including miR-34a, miR-182 and miR-182, are highly expressed in endometrial tumor cells. Studies have shown that restoring the expression of miR-34a, a tumor suppressor miRNA, can prevent cell proliferation, induce apoptosis, and suppress tumor growth in experimental EC models [186]. Additionally, inhibiting the expression of miR-182 suppresses cell proliferation, migration, and invasion, while inhibiting miR-21 expression can enhance apoptosis and increase the sensitivity of endometrial tumor cells to chemotherapy. Ongoing preclinical and clinical studies are exploring the use of miR-21 inhibitors alone or in combination with standard therapies [187,188]. However, it is important to acknowledge that while miRNA-based therapies show promise, further research is needed to optimize their efficacy, safety, and clinical utility in the treatment of EC.

In conclusion, targeted therapy drugs are commonly used alongside additional treatments such as chemotherapy, hormonal therapy, or radiation therapy, depending on the specific cancer characteristics and individual patient needs. The selection of targeted therapy drugs and treatment plans is based on biomarker testing results, tumor characteristics, and clinical trial data. The aim is to create a personalized treatment plan that provides the best possible outcome for the patient.

Ongoing research may lead to the approval of new targeted therapy drugs for treating endometrial cancer. Clinical trials provide an opportunity to evaluate new drugs, treatment combinations, and strategies that are not yet widely accessible.

## 5. Conclusions

This review describes the complexity of endometrial cancers, including various molecular mechanisms, a series of biomarkers, and their critical role in guiding targeted therapies. Multiple subclones in a single tumor can complicate treatment, as different subclones may respond differently to therapies. Therefore, knowing the genomic and epigenomic profile of tumors is essential in guiding treatment plans. Also, combining targeted therapies based on molecular profiles (e.g., PI3K inhibitors with hormone therapy in the CNL subtype) may improve treatment efficacy and overcome resistance mechanisms. In addition, treatment plans can be monitored based on changes in biomarkers and emerging resistance patterns to optimize therapeutic outcomes. Thus, understanding this complex landscape enables the development of personalized treatment strategies and opens up new future research directions in order to improve EC management and increase the quality of life of patients.

## Figures and Tables

**Table 1:** Therapeutic combinations strategies in endometrial cancer.

Therapy Combinations Strategies	Therapeutical Agents	Refs.
Immunotherapy + Radiotherapy	Pembrolizumab after radiotherapy	[175]
Immunotherapy + Chemotherapy	Pembrolizumab + Doxorubicin	[176]
	Pembrolizumab + Paclitaxel + Carboplatin	[177]
Radiotherapy + Chemotherapy + Immunotherapy	Radiotherapy + Carboplatin or Paclitaxel + Bevacizumab	[138,178]
Immunotherapy + Anti-angiogenic therapy	Pembrolizumab + Lenvatinib	[179,180]
Chemotherapy + Immunotherapy + Anti-angiogenic therapy	Cisplatin + Pembrolizumab + Lenvatinib	[152]
Immunotherapy + PAPP inhibitors	Olaparib + Durvalumab	[181,182]
Anti-Her2 Therapy + Chemotherapy	Trastuzumab + Carboplatin + Paclitaxel	[183]
